# Tumor Necrosis Factor α Influences Phenotypic Plasticity and Promotes Epigenetic Changes in Human Basal Forebrain Cholinergic Neuroblasts

**DOI:** 10.3390/ijms21176128

**Published:** 2020-08-25

**Authors:** Giulia Guarnieri, Erica Sarchielli, Paolo Comeglio, Erika Herrera-Puerta, Irene Piaceri, Benedetta Nacmias, Matteo Benelli, Gavin Kelsey, Mario Maggi, Pasquale Gallina, Gabriella Barbara Vannelli, Annamaria Morelli

**Affiliations:** 1 Section of Human Anatomy and Histology, Department of Experimental and Clinical Medicine, University of Florence, 50134 Florence, Italy; erica.sarchielli@unifi.it (E.S.); vannelli@unifi.it (G.B.V.); 2 Sexual Medicine and Andrology Unit, Department of Experimental and Clinical Biomedical Sciences “Mario Serio”, University of Florence, 50134 Florence, Italy; paolo.comeglio@unifi.it; 3 Biology Group CES-EIA, CES University, Medellín 050021, Colombia; yamile.herrera@gmail.com; 4 Department of Neuroscience, Psychology, Drug Research and Child Health, University of Florence, 50134 Florence, Italy; irene.piaceri@gmail.com (I.P.); benedetta.nacmias@unifi.it (B.N.); 5 Bioinformatics Unit, Hospital of Prato, Azienda USL Toscana Centro, 50122 Prato, Italy; matteo.benelli@uslcentro.toscana.it; 6 Epigenetics Programme, The Babraham Institute, Cambridge CB22 3AT, UK; gavin.kelsey@babraham.ac.uk; 7 Centre for Trophoblast Research, University of Cambridge, Cambridge CB2 1TN, UK; 8 Endocrinology Unit, Department of Experimental and Clinical Biomedical Sciences “Mario Serio”, University of Florence, 50134 Florence, Italy; mario.maggi@unifi.it; 9 Neurosurgical Unit, Department of Neurosciences, Psychology, Drug Research and Child Health, University of Florence, 50134 Florence, Italy; pgallina@unifi.it

**Keywords:** neuroinflammation, Alzheimer’s disease, neurogenesis, human fetal neurons, DNA methylation, nucleus basalis of Meynert, ciliogenesis, NGF, TNFα receptors

## Abstract

TNFα is the main proinflammatory cytokine implicated in the pathogenesis of neurodegenerative disorders, but it also modulates physiological functions in both the developing and adult brain. In this study, we investigated a potential direct role of TNFα in determining phenotypic changes of a recently established cellular model of human basal forebrain cholinergic neuroblasts isolated from the nucleus basalis of Meynert (hfNBMs). Exposing hfNBMs to TNFα reduced the expression of immature markers, such as nestin and β-tubulin III, and inhibited primary cilium formation. On the contrary, TNFα increased the expression of TNFα receptor TNFR2 and the mature neuron marker MAP2, also promoting neurite elongation. Moreover, TNFα affected nerve growth factor receptor expression. We also found that TNFα induced the expression of DNA-methylation enzymes and, accordingly, downregulated genes involved in neuronal development through epigenetic mechanisms, as demonstrated by methylome analysis. In summary, TNFα showed a dual role on hfNBMs phenotypic plasticity, exerting a negative influence on neurogenesis despite a positive effect on differentiation, through mechanisms that remain to be elucidated. Our results help to clarify the complexity of TNFα effects in human neurons and suggest that manipulation of TNFα signaling could provide a potential therapeutic approach against neurodegenerative disorders.

## 1. Introduction

The term neuroinflammation is currently used to indicate a cytokine-mediated inflammatory response that originates in the central nervous system (CNS) by the regulation of different cell types, including immune and neuronal cells, to protect the brain from local and peripheral insults. However, when a neuroinflammatory response persists, it may trigger dangerous processes for CNS health. During the past years, growing evidence has shown that chronic inflammatory conditions play a crucial role in the pathogenic mechanisms underlying the onset and progression of neurodegenerative disorders, especially those characterized by cognitive decline, such as Alzheimer’s disease (AD) and Parkinson’s disease (PD) [1]. Epidemiological studies and research in animal models indicate that cognitive and memory impairments are strictly linked to altered production of inflammatory mediators [2,3,4]. Accordingly, anti-inflammatory drugs seem to protect against the development of neurodegeneration and cognitive symptoms [5,6,7]. On the other hand, it has been demonstrated that pro-inflammatory pathways may affect tissue organization and higher brain function during prenatal and postnatal periods [8]. 

Among the pro-inflammatory mediators, tumor necrosis factor α (TNFα) represents one of the most important pleiotropic cytokines involved in the neuroinflammatory response. Accumulating evidence has indicated that, under physiological conditions, TNFα exerts crucial biological functions both in the developing and adult brain but, in other conditions the chronic production of the cytokine causes neurotoxic effects, leading to neurodegeneration [9]. 

Cytokines, such as TNFα can pass the placenta-fetal barrier reaching the fetal brain and influencing its development [10]. In particular, high level of TNFα during the prenatal period has been associated with alteration in cognitive development in the postnatal and childhood life [8]. In addition, accumulating evidence have shown that TNFα negatively affects fetal and adult neurogenesis [11]. Indeed, in vitro studies revealed that TNFα exposure reduces the number of neuronal progenitors in embryonic rat whole-brain neurospheres [12] and in rat as well as mouse hippocampal progenitor cells [13,14,15]. Likewise, TNFα impaired neurogenesis in human striatal and hippocampal immortalized cell lines [16]. 

Basal forebrain cholinergic neurons (BFCNs) are magnocellular neurons present in the medial and ventral cerebral hemisphere and organized in distinctive cellular structures. Among these structures, the nucleus basalis of Meynert (NBM) includes neurons that provide the major cholinergic input to the entire neocortex, hippocampus and amygdala, thus occupying a crucial position for modulating cognitive functions, such as attention, learning and memory [17]. The selective degeneration of BFCNs, especially those located in the NBM, represents a pathological hallmark for AD patients [18] and has a predictive value for cognitive deficits in PD patients [19]. An excessive production of TNFα has been associated with cognitive decline [6]. Indeed, a role of TNFα in potentiating cell death of NBM cholinergic neurons, possibly via retrograde axonal damage, has been demonstrated both in vitro and in vivo in the rat [20]. However, the TNFα effects on human BFCN development and plasticity remain to be clarified. Indeed, the mechanisms through which pro-inflammatory mediators affect human brain structures are complex and difficult to be elucidated, especially due to the limited availability of study models for both in vivo and in vitro investigations.

Concerning the molecular mechanisms through which TNFα could affect neuronal development and function, several lines of evidence suggested that environmental cell-extrinsic cues, such as cytokine signaling, may regulate differentiation plasticity of neural cells and neural circuits through the involvement of cell-intrinsic epigenetic mechanisms [21,22,23]. Epigenetic modifications, particularly DNA methylation, have been implicated in mammalian brain development and function, both during pre-/peri-natal CNS maturation and in the adult brain [24,25]. Moreover, an altered DNA methylation pattern has been demonstrated in neurodegenerative diseases, such as AD and PD [26], which are typically characterized by neuroinflammation and a predominant presence of TNFα cytokine. Interestingly, DNA methylation changes and other epigenetic mechanisms underlying TNFα-mediated effects have been reported in a number of cell types [27,28,29,30,31,32,33]. Overall, these findings suggest the importance of analyzing epigenetic actions of TNFα in human neuronal models.

Because of the obvious limitations of study methods that can be used in living humans, the availability of an in vitro model represents a valuable tool in this context. In this work, we took advantage of a primary culture of cholinergic neuroblasts isolated from the human fetal NBM (hfNBMs), which we previously characterized [34,35,36], in order to investigate the direct effects of TNFα on phenotypic plasticity of human NBM cholinergic neurons. Indeed, fetal tissue is a rich source of cells already committed toward a specific cell fate, although they retain immature features and, therefore, are able to proliferate and fully maturate in vitro. Hence, such a model is useful for exploring simultaneously different effects, including those related to neurogenesis and cellular plasticity, as well as those related to more differentiated aspects. In addition, here we evaluated, for the first time, the DNA methylation status of hfNBMs under TNFα stimulation by genome-wide analysis.

## 2. Results

### 2.1. TNFα Affects hfNBM Cell Phenotype

**Figure 1:**
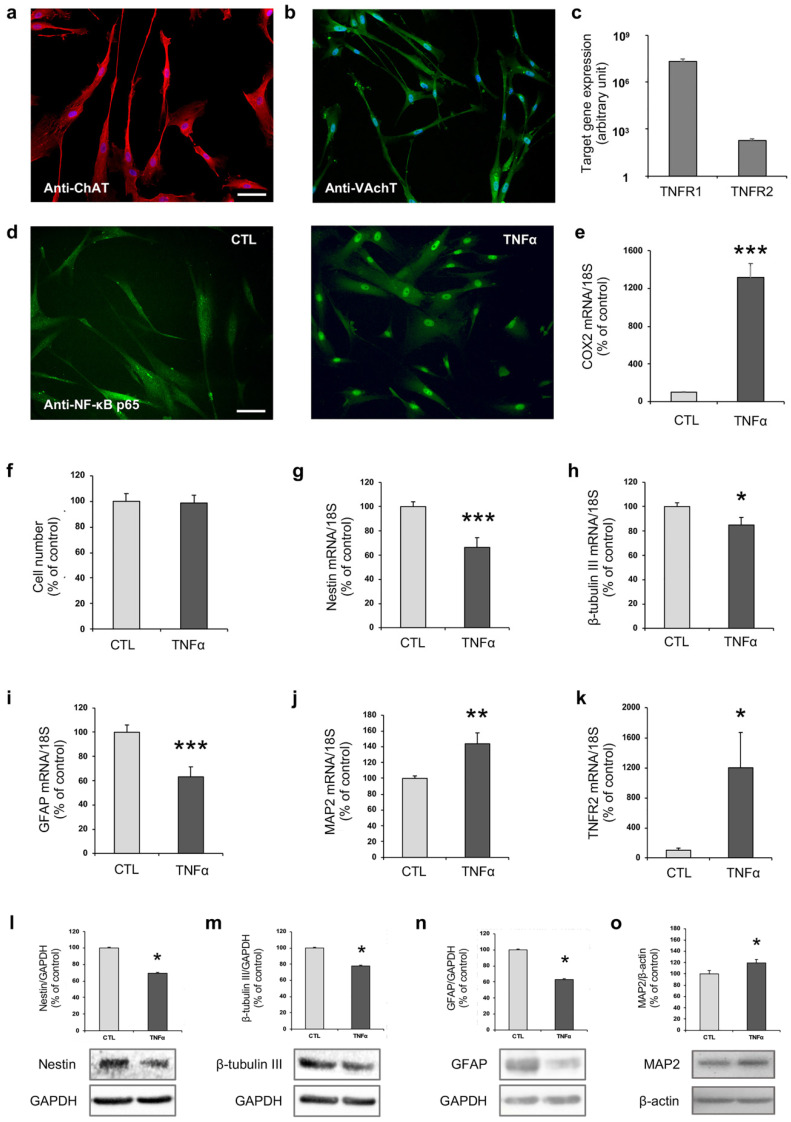
TNFα affects hfNBM cell phenotype. (**a**,**b**) Representative images showing (**a**) ChAT and (**b**) VAchT expression in hfNBMs as evaluated by immunofluorescence analysis (DAPI counterstained nuclei, scale bar 100 µm). (**c**) Relative mRNA expression by qRT-PCR analysis of TNFR1 and TNFR2 receptors normalized over 18S ribosomal subunit, taken as reference gene, and reported as mean ± SEM (*n* = 6). (**d**) Immunofluorescent analysis of NF-κB p65 nuclear translocation after TNFα stimulus (10 ng/mL, 3 h) in comparison to untreated cells (CTL; scale bar 100 µm). (**e**) COX2 mRNA expression in hfNBMs by qRT-PCR after TNFα stimulation (10 ng/mL, 24 h). Data are normalized over 18S ribosomal RNA subunit and reported as percentage of untreated cells (CTL) and displayed as mean ± SEM of three separate experiments performed in triplicate (unpaired Student’s *t*-test; *** *p* < 0.001 vs. CTL; *n* = 9). (**f**) MTT analysis of hfNBMs treated or not (CTL) with TNFα (10 ng/mL, 24 h; *n* = 3). (**g**) nestin, (**h**) β-tubulin III, (**i**) GFAP, (**j**) MAP2 and (**k**) TNFR2 mRNA expression in untreated (CTL) and TNFα-treated (10 ng/mL, 24 h) hfNBMs by qRT-PCR. Data are normalized over 18S ribosomal RNA subunit and reported as percentage of CTL and displayed as mean ±S EM of four separate experiments performed in triplicate (unpaired Student’s *t*-test; *** *p* < 0.001, ** *p* < 0.01, * *p* < 0.05 vs. CTL; *n* = 12 or *n* = 6). (**l**) nestin, (**m**) β-tubulin III, (**n**) GFAP and (**o**) MAP2 protein expression in untreated (CTL) and TNFα-treated (10 ng/mL, 24 h) hfNBMs by Western blot analysis. Band intensity of the specific protein was normalized over GAPDH or β-actin signal, expressed as percentage of CTL and displayed as mean ± SEM of three separate experiments (unpaired Student’s *t*-test; * *p* < 0.05 vs. CTL; *n* = 3).

The phenotype of hfNBMs and their ability to respond to TNFα were confirmed as already published [35,36]. In particular, the cholinergic phenotype of hfNBMs is shown in Figure 1 by the strong immunopositivity to choline acetyltransferase (ChAT; Figure 1a), the enzyme essential for acetylcholine (Ach) synthesis, and to the specific Ach vesicular transporter (VAchT; Figure 1b). Moreover, hfNBMs express both types of TNFα receptors (*TNFR1* and *TNFR2*), as detected by quantitative real time RT-PCR (qRT-PCR; Figure 1c), being *TNFR1* more abundant than *TNFR2*. In addition, as reported in Figure 1f, we verified that 24-h exposure of TNFα (10 ng/mL) did not affect cell viability. Next, NF-κB p65 immunolocalization was used as an indicator of TNFα-induced inflammatory response. As shown in Figure 1d, untreated cells retained NF-κB p65 in the cytoplasm in an inactive form, with a very low percentage of cells showing nuclear positivity (1.48 ± 0.99%). Exposing hfNBMs to 10 ng/mL TNFα for 3 h induced a significant nuclear translocation of NF-κB p65 (86.16 ± 2.03%; *p* < 0.0001). Accordingly, prolonged exposure to TNFα (10 ng/mL, 24 h) significantly increased the mRNA expression of the NF-κB p65 target gene *COX2* (cyclooxygenase 2; *p* < 0.001; Figure 1e). 

As we previously reported [35], hfNBMs express high mRNA levels of the neural precursor marker nestin, as well as the neuronal progenitor marker β-tubulin ΙΙΙ, indicating the presence of cells exhibiting immature features. Interestingly, as detected by qRT-PCR, exposing hfNBMs to TNFα (10 ng/mL; 24 h) significantly decreased the mRNA expression of nestin (*p* < 0.001; Figure 1g), as well as well as β-tubulin III (*p* < 0.05; Figure 1h), in comparison with unstimulated cells. In addition, the mRNA levels of *GFAP* (glial fibrillary acidic protein), a specific marker for the glial lineage also previously detected in these cells, even at low levels [35], were significantly reduced after TNFα treatment (*p* < 0.001; Figure 1i). In contrast, TNFα stimulation significantly increased mRNA expression of the mature neuron marker microtubule associate protein 2 (MAP2) (*p* < 0.01; Figure 1j). The effect of TNFα on immature and mature neural/neuronal markers nestin, β-tubulin III, GFAP, and MAP2 was also confirmed in terms of protein expression (Figure 1l–o, respectively). Interestingly, TNFα treatment determined a one log unit increase of *TNFR2* mRNA expression (*p* < 0.05; Figure 1k). 

**Figure 2:**
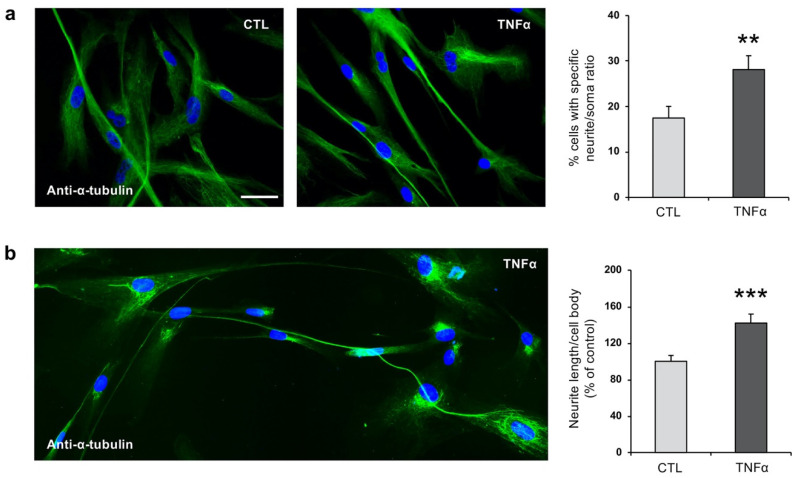
TNFα increases neurite outgrowth in hfNBMs. (**a**,**b**) Representative images (left) and graph (right) showing (**a**) neurite outgrowth count and (**b**) length by α-tubulin staining in untreated (CTL) or TNFα-treated (10 ng/mL, 24 h) hfNBMs (DAPI counterstained nuclei, scale bar 50 µm). Bar graph in panel **a** shows the percentage of cells with neurites longer than four times the cell body calculated by counting ten fields per slide of three separate experiments (unpaired Student’s *t*-test; ** *p* < 0.01 vs. CTL; *n* = 30). Bar graph in panel **b** shows the ratio between neurite length and cell body diameter calculated using ImageJ NeuronJ plugin in ten different fields for each condition, expressed as percentage of CTL and displayed as mean ± SEM of three separate experiments (unpaired Student’s *t*-test; *** *p* < 0.001 vs. CTL; *n* = 30).

To further study the effect of TNFα on the phenotype of hfNBMs, we next analyzed neurite outgrowth. Serum-starved cells were treated with 10 ng/mL TNFα for 24 h, and the presence of neurite elongation was evaluated by immunofluorescent detection of α-tubulin, in comparison with untreated cells. The immunofluorescent analysis showed about 18% of cells (17.46 ± 2.56%) with neurites longer than four times the cell body in untreated cells, while the percentage was significantly increased by TNFα treatment (28.11 ± 3.05%, *p* < 0.01; Figure 2a). Most notably, the mean neurite length of TNFα stimulated cells was significantly increased, by 40% as compared to unstimulated cells (*p* < 0.001; Figure 2b).

### 2.2. TNFα Impairs Ciliogenesis in hfNBMs

**Figure 3:**
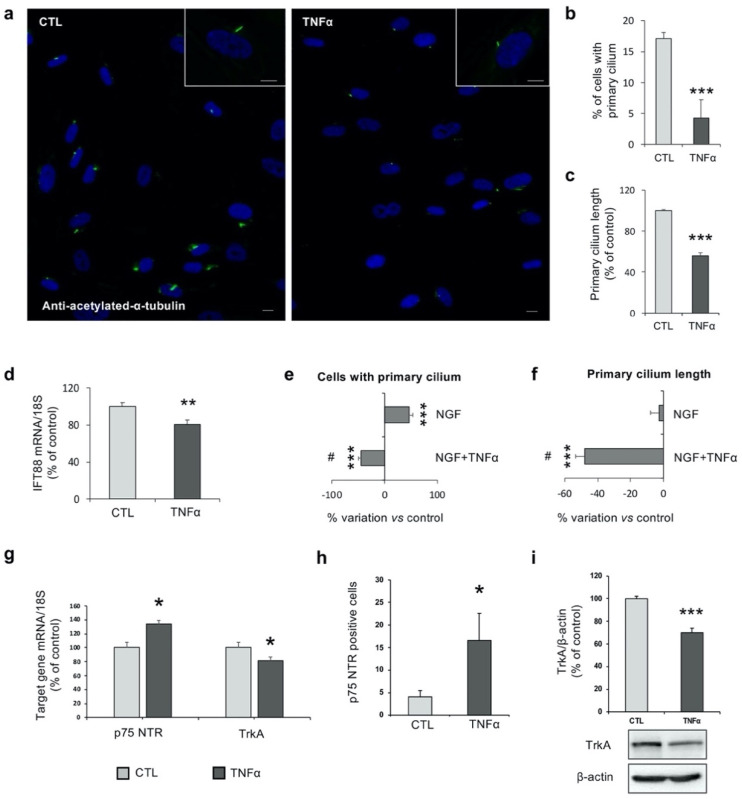
TNFα inhibits primary cilium formation in hfNBMs. (**a**) Representative images showing the primary cilium in untreated (CTL) or TNFα-treated (10 ng/mL, 24 h) hfNBMs by immunofluorescence using the specific marker acetylated α-tubulin (DAPI counterstained nuclei; scale bars 10 µm). (**b**) Number of cells with a primary cilium counted in ten different fields for each condition and expressed as percentage of DAPI-stained (blue) total cells. Results are reported as mean ± SEM of three separate experiments (unpaired Student’s *t*-test; *** *p* < 0.001 vs. CTL; *n* = 30). (**c**) Evaluation of the primary cilium length performed in at least 15 cells for untreated (CTL) and TNFα-treated (10 ng/mL, 24 h) hfNBMs, using ImageJ NeuronJ plugin. Data are expressed as percentage of CTL and reported as mean ± SEM of three separate experiments (unpaired Student’s *t*-test; *** *p* < 0.001 vs. CTL; *n* = 15). (**d**) IFT88 mRNA expression in untreated (CTL) and TNFα-treated (10 ng/mL, 24 h) hfNBMs by qRT-PCR. Data are normalized over 18S ribosomal RNA subunit and reported as percentage of CTL and displayed as mean ± SEM of four separate experiments performed in triplicate (unpaired Student’s *t*-test; ** *p* < 0.01 vs. CTL; *n* = 12). (**e**) Number of hfNBMs with a primary cilium after NGF treatment (100 ng/mL) in presence (NGF+TNFα) or absence (NGF) of TNFα (10 ng/mL) for 24 h. Data are reported as percentage of variation of untreated cells (CTL) and displayed as mean ± SEM of four separate experiments (one-way ANOVA followed by Tukey’s post hoc analysis; *** *p* < 0.001 vs. CTL; ^#^ *p* < 0.001 vs. NGF; *n* = 40). (**f**) Evaluation of the primary cilium length performed in at least 15 hfNBMs for NGF-treated cells (100 ng/mL) in presence (NGF+TNFα) or absence (NGF) of TNFα (10 ng/mL) for 24 h. Data are calculated using ImageJ, reported as percentage of variation of untreated cells (CTL) and displayed as mean ± SEM of three separate experiments (one-way ANOVA followed by Tukey’s post hoc analysis; *** *p* < 0.001 vs. CTL; ^#^ *p* < 0.001 vs. NGF; *n* = 15). (**g**) (left) p75 NTR and (right) TrkA mRNA expression in untreated (CTL) and TNFα-treated (10 ng/mL, 24 h) hfNBMs by qRT-PCR. Data are normalized over 18S ribosomal RNA subunit and reported as percentage of CTL and displayed as mean ± SEM of three separate experiments performed in triplicate (unpaired Student’s *t*-test; * *p* < 0.05 vs. CTL; *n* = 12). (**h**) Flow cytometric analysis for p75 NTR protein in untreated (CTL) and TNFα-treated (10 ng/mL, 24 h) hfNBMs. Data represent the percentage of positive cells reported as mean ± SEM of three separate experiments performed in duplicate (unpaired Student’s *t*-test (* *p* < 0.05 vs. CTL; *n* = 6). (**i**) TrkA protein expression in untreated (CTL) and TNFα-treated (10 ng/mL, 24 h) hfNBMs by Western blot analysis. Bands intensity are normalized over β-actin signal, expressed as percentage of CTL and displayed as mean ± SEM of three separate experiments performed in duplicate (unpaired Student’s *t*-test; *** *p* < 0.001 vs. CTL; *n* = 6).

As previously reported [35], hfNBMs express a primary cilium. Immunofluorescence analysis using the specific marker acetylated α-tubulin revealed the presence of a primary cilium in basal conditions in about 20% of hfNBMs (Figure 3a), while TNFα exposure (10 ng/mL for 24 h) caused a significant reduction of the percentage of cells exhibiting a primary cilium (4.29 ± 2.03%, *p* < 0.001; Figure 3b), as well as a significant reduction of the primary cilium length (*p* < 0.001; Figure 3c). Moreover, qRT-PCR analysis showed that the mRNA expression of *IFT88 (intraflagellar Transport 88)*, an intraflagellar transporter specific for primary-cilium formation, was significantly reduced by TNFα treatment (*p* < 0.05; Figure 3d). Since we previously demonstrated that nerve growth factor (NGF) induced ciliogenesis in hfNBMs [35], we investigated whether TNFα could interfere with this effect. Interestingly, TNFα exposure abolished the positive effect of NGF (100 ng/mL; *p* < 0.001 NGF vs. untreated cells) on ciliogenesis in terms of percentage of ciliated cells (*p* < 0.001 vs. untreated cells and *p* < 0.001 vs. NGF; Figure 3e). In addition, NGF did not change the primary cilium length either alone or in the presence of TNFα treatment (Figure 3f). 

Given the effects of TNFα in affecting NGF-induced ciliogenesis, we evaluated whether the cytokine could affect NGF signaling. As shown in Figure 3g, TNFα exposure (10 ng/mL for 24 h) significantly increased the mRNA expression of *p75 NTR (neurotrophin Receptor p75)*, the low affinity NGF receptor, but reduced expression of the high affinity NGF receptor *TrkA* (tropomyosin receptor kinase A; *p* < 0.05 vs. untreated cells). These results were then confirmed by protein expression analysis (Figure 3h,i). Indeed, flow cytometry experiments using specific antibodies evidenced a low basal expression of p75 NTR (5.05 ± 4.72%), while TNFα treatment (10 ng/mL for 24 h) significantly increased the percentage of p75 NTR-positive cells (23.13 ± 9.47%, *p* < 0.05; Figure 3h). On the contrary, although no significant change was detected in terms of number of TrkA-positive cells by flow cytometry (data not shown), Western blot analysis showed a significant reduction of TrkA protein expression following TNFα stimulation (*p* < 0.001; Figure 3i).

### 2.3. TNFα Modulates the Expression of Enzymes Involved in DNA Methylation

**Figure 4:**
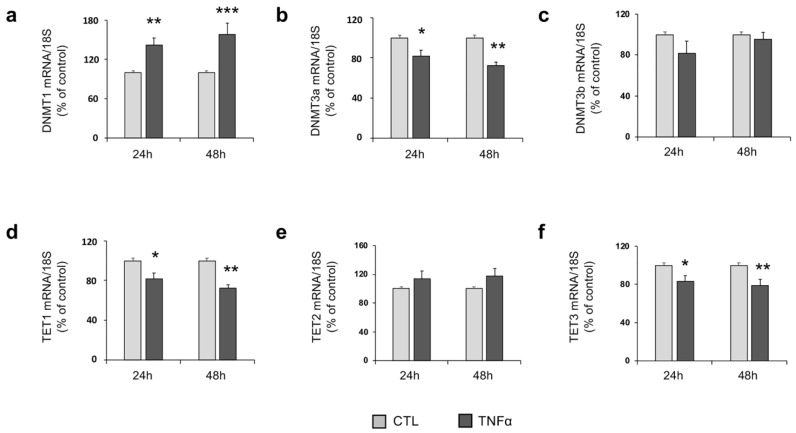
TNFα affects DNA Methyltransferases (DNMTs) and Ten Eleven Translocation (TETs) mRNA levels. (**a**) DNMT1, (**b**) DNMT3a, (**c**) DNMT3b, (**d**) TET1, (**e**) TET2 and (**f**) TET3 mRNA expression in untreated (CTL) and TNFα-treated (10 ng/mL) hfNBMs at 24- (left) and 48-h (right) of stimulation, as detected by qRT-PCR. Data are normalized over 18S ribosomal RNA subunit and reported as percentage of CTL and displayed as mean ± SEM of three separate experiments performed in triplicate (unpaired Student’s *t*-test; *** *p* < 0.0001, ** *p* < 0.01, * *p* < 0.05 vs. CTL; *n* = 9).

To explore the role of TNFα in epigenetic mechanisms in hfNBMs, we firstly investigated the effects of the cytokine on the expression of key enzymes involved in DNA methylation, including DNA methyltransferases (DNMT1, DNMT3a, DNMT3b) and ten-eleven translocation enzymes (TET1, TET2, TET3). As shown in Figure 4, TNFα treatment (10 ng/mL), at both 24- and 48-h time points, significantly increased the mRNA expression of *DNMT1* (*p* < 0.01 and *p* < 0.001, respectively; Figure 4a), the principal maintenance DNA methyltransferase, and the de novo activity *DNTMT3a* (*p* < 0.05 and *p* < 0.01, respectively; Figure 4b) mRNA expression. No significant changes were observed in mRNA abundance of *DNMT3b* (Figure 4c). Regarding the demethylation enzymes, TNFα treatment resulted in a significant decrease in both *TET1* (*p* < 0.05 at 24 h; *p* < 0.01 at 48 h; Figure 4d) and *TET3* (*p* < 0.05 at 24 h; *p* < 0.01 at 48 h; Figure 4f) mRNA expression at both time points, without significant changes in *TET2* mRNA levels (Figure 4e). Moreover, further analysis at the protein level confirmed the significant increase in DNMT1 expression upon 24-h TNFα stimulation (*p* < 0.05; Supplementary Figure S1).

### 2.4. TNFα Changes DNA Methylation of Target Regulatory Elements in hfNBMs

Based on the results indicating the effects of TNFα in regulating enzymes involved in DNA methylation mechanisms, we hypothesized a contribution of the cytokine to mediating epigenetic changes in hfNBMs. To determine whether TNFα affected DNA methylation status of regulatory regions, a genome-wide DNA methylation analysis of TNFα-treated (10 ng/mL for 24 and 48 h) and untreated hfNBMs by reduced representative bisulfite sequencing (RRBS) was performed using three biological replicates per condition and time point. Overall methylation of the CpG sites assessed by RRBS in cultured hfNBMs ranged from 81.8% to 84.2% and from 80.8% to 81.82% at 24 and 48 h, respectively (Supplementary Table S3). Similarly, the global methylation profile of TNFα-treated cells showed a range from 81.9% to 83.3% for 24 h and 81.1% to 84.4% for 48 h of treatment, but with no significant difference compared to untreated cells. The very low rate of methylated cytosines in non-CpG contexts (0.4–0.8% across all samples, where “C” is located in “CHH” or “CHG” regions - H=A, T or C – Supplementary Table S3), indicates a uniformly high bisulfite conversion rate (>99%).

**Figure 5:**
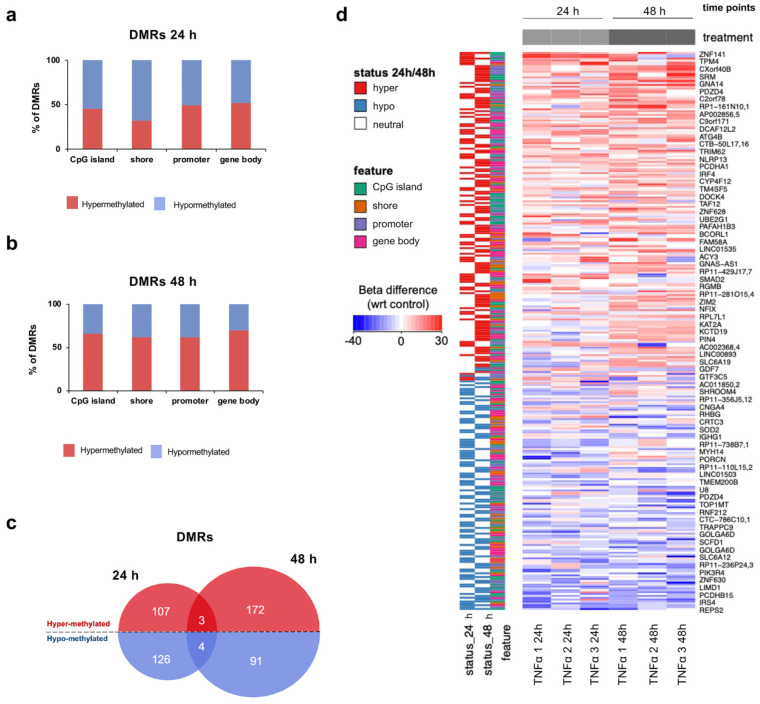
Characteristics of regions differentially methylated by TNFα treatment. (**a**,**b**) Percentage of hyper-methylation (red) and hypo-methylation (blue) of significant differentially methylated genomic elements (CpG island, shore, promoter, gene body) at (**a**) 24 h and (**b**) 48 h of TNFα stimulation in comparison to untreated samples. Data are expressed as percentage of total number of significant differentially methylated regions (DMRs; FDR <0.05 logistic regression corrected for multiple comparisons using Benjamini-Hochberg, methylation difference ≥10%). (**c**) Venn diagram reporting the number of hyper- and hypo-methylated significant DMRs (FDR <0.05 logistic regression corrected for multiple comparisons using Benjamini-Hochberg, methylation difference ≥10%) detected at 24- and 48- hour time-points. (**d**) Heat map showing the difference of methylation level of TNFα-treated hfNBMs with respect to untreated cells at 24- and 48- hour time-points. Shown are 24 h and 48 h DMRs evaluated at CpG islands (green), shores (orange), promoters (purple) and gene bodies (pink) and detectable in both time points.

To investigate the DNA methylation changes under inflammatory conditions, we carried out a more detailed analysis of specific genomic annotations, focusing on promoters, CpG islands, shores and gene bodies, as these regions are likely to contain DNA methylation patterns important for gene transcription. Although we observed very similar DNA methylation level in TNFα-treated and untreated cells within all categories analyzed (average of Pearson’s correlation coefficient R=0.989 for both time points; Supplementary Figure S2a,b), we identified 233 statistically differentially methylated regions (DMRs; difference >10% and FDR <0.05) between treated and control hfNBMs at the 24-h time-point (CpG islands, *n* = 66; shores, *n* = 38; promoters, *n* = 57; gene bodies, *n* = 72; Supplementary Figure S2a), and 263 at the 48-h time point (CpG islands, *n* = 74; shores, *n* = 45; promoters, *n* = 68; gene bodies, *n* = 76; Supplementary Figure S2b; Supplementary Table S4). In addition, looking at the proportion of hyper- or hypo-methylation within significantly variable genomic elements, we found that the DMRs at the 24-h time-point, tended to be equally distributed between hyper- (CpG islands: 45.45%; shores: 31.58%; promoters: 49.12%; gene bodies: 51.39%) and hypo-methylation (CpG islands: 54.55%; shores: 68.42%; promoters: 50.88%; gene bodies: 48.61%; Figure 5a), whereas there was an enrichment of hyper-methylation into all DMR categories (CpG islands: 66.22%; shores: 62.22%; promoters: 61.76%; gene bodies: 69.74%) at 48 h of TNFα treatment (Figure 5b). Although only four hypo-methylated and three hyper-methylated significant DMRs (FDR <0.05) overlapped between 24- and 48-h time-points at the >10% change threshold (Figure 5c), we observed a concordance in the trend of the majority of detectable DMRs (Figure 5d). 

### 2.5. TNFα Exposure Changes DNA Methylation of Nervous System Development-Related Genes

**Figure 6:**
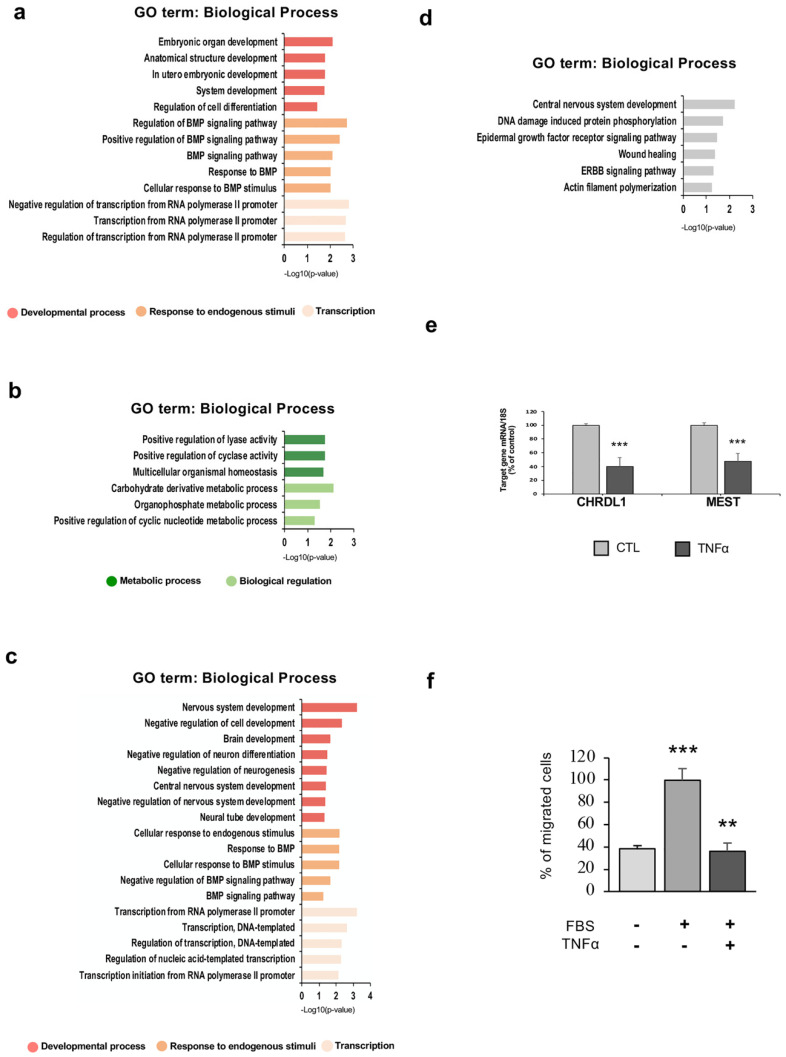
Functional analysis of DNA methylation changes in TNFα-treated hfNBMs. (**a**–**d**) GO-based annotation (GO_Biological_Process) of genes associated to significant (**a**) hypermethylated and (**b**) hypomethylated DMRs at 24 h of TNFα stimulation, and for significant (**c**) hyper-methylated and (**d**) hypo-methylated DMRs at 48 h of TNFα stimulation in comparison to untreated cells (FDR <0.05 logistic regression corrected for multiple comparisons using Benjamini-Hochberg, methylation difference ≥10%). More significant terms for category are shown and data are expressed as -log10 transformed p-value of GO terms (*p* < 0.05). (**e**) CHRDL1 (left) and MEST (right) mRNA expression in untreated (CTL) and TNFα-treated (10 ng/mL, 48 h) hfNBMs, as detected by qRT-PCR. Data are normalized over 18S ribosomal RNA subunit and reported as percentage of CTL and displayed as mean ± SEM of three separate experiments performed in triplicate (unpaired Student’s *t*-test (*** *p* < 0.001 vs. CTL; *n* = 9). (**f**) Bar graph showing quantitative analysis of transwell migration assay of untreated (CTL) and TNFα-treated (10 ng/mL, 48 h) hfNBMs in absence (CTL-Serum-free) or presence (CTL-FBS; TNFα) of 10% FBS-serum as chemoattractant. Data are evaluated in ten different field for each condition, expressed as percentage of migrated cells and displayed as mean ± SEM of three separate experiments (one-way ANOVA followed by Tukey’s post hoc analysis; *** *p* < 0.001 vs. CTL-Serum-free, ** *p* < 0.01 vs. CTL-FBS; *n* = 30).

We next investigated whether the genes annotated to significant differentially methylated genomic elements were involved in specific biological functions. A list of 227 genes (*n* = 104 hyper- and *n* = 123 hypo-methylated) for DMRs at the 24-h time-point and 243 genes (*n* = 156 hyper- and *n* = 87 hypo-methylated) at the 48-h time-point was obtained. Gene ontology (GO) functional analysis using DAVID was performed on genes that contain differentially methylated CpG islands, shores, promoters and gene bodies (difference >10% and FDR <0.05), all clustered in a single dataset. Functional annotation for biological processes at 24 h indicated that genes associated with hyper-methylated elements were enriched in developmental processes, regulation of transcription, and response to endogenous stimuli (Figure 6a). On the other hand, genes related to hypomethylated elements were over-represented in metabolic processes and biological regulation (Figure 6b). Interestingly, GO analysis for the hyper-methylated 48-h gene list revealed an enrichment of genes involved in nervous system development and regulation of transcription, suggesting the potential role of inflammation in interfering with hfNBMs neuronal development (Figure 6c). Lastly, enrichment for the central nervous system development category was also observed for the 48-h hypo-methylated gene list (Figure 6d). 

In order to identify methylation differences of genomic elements (CpG islands, shores, promoters and gene bodies) upon TNFα stimulation that are likely to have the greatest functional outcome, the DMRs were filtered for a significant minimum difference of 20% and the corresponding list of genes is reported in Table 1. A total of 25 and 46 differentially methylated genomic elements, corresponding to nine and 16 genes, were found at the 24- and 48-h time-points, respectively. Interestingly, among variable regions at the 48-h time-point, we found genes involved in nervous system development (*MEST*, *GSC*, *CHRDL1*, *HDAC10*), differentiation (*CHRDL1*, *MYLB2*, *HDAC10*) and migration processes (*MEST* and *CHRDL1*). On the other hand, data from the 24-h time-point revealed the presence of genes involved in heterogeneous biological functions, such as regulation of transcription (*PLAGL1* and *NR0B1*), protein turnover and stabilization (*HSPA12B* and *LAMP2*) and signal transduction (*KSR1* and *NR0B1*).

### 2.6. Hypermethylation of Genes Involved in Nervous System Development and Migration are Associated with a Reduced mRNA Expression After Inflammatory Exposure

To investigate whether the TNFα-induced methylation changes could actually affect gene expression, we performed qRT-PCR in RNA extracts from samples used for DNA methylation studies. The analysis was performed for a set of genes annotated to significant DMRs (difference >20% and FDR <0.05) and selected from those reported in Table 1. A significant change of mRNA expression was found for the chordin like 1 (*CHRDL1*) and mesoderm specific transcript (*MEST*) genes, both with TNFα-induced CpG island hyper-methylation. Indeed, the mRNA levels for these genes were significantly reduced after 48 h of TNFα treatment (*p* < 0.001; Figure 6e). No statistical differences were found for the other genes analyzed (*MYLB2*, *PRMD7*, *HDAC10*, *DCAF12L2*, *PLAGL1*, *KSR1*; data not shown). In order to validate the increase of DNA methylation level of the *CHRDL1* CpG Island (115193-GRch37, UCSC genome) after 48-h TNFα stimulation, a methylation-sensitive restriction qPCR analysis was performed using the EpiTect Methyl II PCR assay. Serum-starved hfNBMs were treated with 10 ng/mL TNFα for 48 h and analyzed in comparison with untreated cells. As shown in Supplementary Figure S3, a significant 2.5-fold increase of methylation status was detected in 48-h TNFα treated cells in comparison with untreated cells (*p* < 0.05), confirming the hyper-methylation of this region. 

### 2.7. hfNBM Cell Migration is Inhibited by TNFα Stimulation

Since we found a significant downregulation of genes such as CHRDL1 and MEST that play an important role in cell migration, we next analyzed, from a functional point of view, whether TNFα affected the migratory properties of hfNBMs. Using a Boyden chamber-based migration assay, serum-starved hfNBMs, with or without 48-h TNFα (10 ng/mL) pre-treatment, were seeded in the upper chamber and migration was stimulated by 10% fetal bovine serum (FBS), added in the lower well of the chamber, which is generally used for this purpose [37]. Serum-free medium in the lower well was used as negative control. As shown in Figure 6f, 10% FBS significantly increased the number of migrated cells (*p* < 0.001 vs. FBS-free medium), while TNFα pre-treatment completely prevented this effect.

## 3. Discussion

In this study, we tested the direct action of TNFα in human NBM cholinergic neurons from the fetal brain. The overall conclusions are that the cytokine may exert pleiotropic effects in these cells, interfering with their differentiation potential and phenotypic plasticity. Of note, epigenetic mechanisms appeared to be implicated in these actions, as suggested by our observation of TNFα-induced changes in DNA methylation status of target genes. Our results may contribute to improve the knowledge about the complexity of TNFα effects in contributing to CNS diseases.

As previously described [35], hfNBMs possess features indicative of cholinergic identity, as they express specific markers, including ChAT and VAchT, respond to the main neurotrophic factor for this neuronal population (NGF) and are able to release Ach in the culture medium. Interestingly, when injected in NBM-lesioned rats, hfNBMs promoted functional effects most likely because of an improvement of the cholinergic signaling [35]. Being of fetal origin, hfNBMs also showed immature features since they expressed precursor neuronal markers (nestin, β-tubulin III) along with a primary cilium, which is known to be implicated in neuro-developmental processes [38]. Moreover, the cell culture retained the same phenotype for several passages in culture. Hence, the cellular model used in this study represents a valid tool to investigate how inflammatory insults may negatively impact on human NBM cholinergic neurons. 

It has been reported that TNFα impairs fetal and adult neurogenesis [11,14]. Indeed, in vitro studies revealed that TNFα exposure reduces the number of neuronal progenitors in embryonic rat whole-brain neurospheres [12] and in rat and mouse hippocampal progenitor cells [13,14,15]. In addition, it impairs neurogenesis in human striatal and hippocampal cell lines [16]. On the other hand, TNFα has also been reported to induce neuronal differentiation of neural progenitor cells (NPCs) in mice [13] and proliferation of neural stem cells (NSCs) from the subventricular zone of adult rats [39]. However, very little is known about the effect of TNFα on cell plasticity and differentiation of human neuronal precursors. Using human fetal cortical progenitors, Peng and colleagues demonstrated a reduction in neuronal differentiation and an increase of gliogenesis [40]. In our study we observed that exposing hfNBMs to TNFα determined a significant reduction of both nestin and β-tubulin III expression, indicating a detrimental effect on the immature properties of the primary cell culture. In addition, we found that TNFα interfered with the formation of the primary cilium in hfNBMs. These results are consistent with similar findings described in different cellular types, such as human fetal hypothalamic neurons [41] and mouse mesenchymal stromal cells [42] where TNFα inhibited ciliogenesis. Primary cilia are non-motile, sensory antennas protruding 4–8 µm from the surface of the majority of human cells, including neurons, able to mediate the cellular response to extracellular signals growth and other stimuli [43]. Although many of primary cilium functions remain unclear, it has been recently shown an essential role in neurogenesis, since this organelle regulates specific neurodevelopmental signals, such as sonic hedgehog (Shh), WNT and mitogenic pathways [44]. The TNFα-mediated impairment of ciliogenesis in hfNBMs could be responsible for alterations in neurogenic mechanisms. Indeed, recent studies have implicated primary cilia formation in the mechanism regulating neurogenesis both during fetal development [45] and in the adult brain [46]. Furthermore, we previously described a positive effect of NGF on ciliogenesis in hfNBMs [35], thus supporting a role of this organelle in the NGF-driven NBM neuron maturation. Since TNFα abrogated the effect of NGF on primary cilium formation, we may hypothesize that, under inflammatory conditions, the response of hfNBMs to NGF, and thereby their correct maturation, could be altered. In line with this hypothesis, TNFα significantly decreased the expression of TrkA receptor, which is known to regulate the neurotrophic effect of NGF for NBM neurons [47], thus suggesting that a possible TNFα-mediated mechanism of action is a desensitization to NGF effects in hfNBMs. This hypothesis is in agreement with studies conducted in post-mortem brains of AD patients that revealed a dramatic reduction in the expression of TrkA within NBM [48]. On the other hand, an increase of cells expressing p75 NTR, the low affinity NGF receptor that generally mediates pro-apoptotic signals, was also observed under TNFα stimulation in hfNBMs, thus suggesting a shift in the balance of neuroprotective and neurotoxic pathways mediated by NGF receptors. In addition, deficits in migration, an essential mechanism for neuronal differentiation that allows neurons to reach the proper final destination within the brain, were observed in hfNBMs treated with TNFα. Interestingly, primary cilium has been identified as an essential director of neuronal migration during neurogenic processes [49]. Hence, the TNFα-mediated impairment of primary cilium formation may be responsible for defects of migration ability. Further investigations will clarify the role of primary cilia in hfNBM cell migration.

In contrast to these negative actions, our study also showed a pro-differentiation effect of TNFα on hfNBMs, as indicated by the increased expression of the mature neuron marker MAP2 and the promotion of neurite outgrowth after TNFα treatment. Indeed, the cytokine significantly increased the number of neurons showing neurites and, most notably, it increased neurite length by 40% as compared to untreated cells. Accordingly, a positive effect of the cytokine in mediating neurite outgrowth has been observed in cultured adult sensory rat neurons [50] and in mouse organotypic brain slices [51]. This occurrence may be explained by the recruitment of compensatory mechanisms that allow the cells to resist and survive in response to insults. In this regard, TNFα-treated hfNBMs showed a significant decrease in the mRNA expression of the *GFAP*, a glial-marker also known to be expressed by early stage neural progenitor cells [52]. This effect has already been reported in several studies [53,54,55] and has been associated with glial remodeling processes to ensure neurite outgrowth [56]. Overall, our findings suggest a dual action of TNFα-mediated inflammation on hfNBMs, showing both beneficial and detrimental effects on the distinct components of the primary culture. In particular, TNFα appeared to affect the neuronal plasticity of hfNBMs, acting on the immature properties of the culture, as demonstrated by the marked reduction of nestin, β-tubulin III, and *GFAP* expression and interfering with neurogenic mechanisms, including primary cilium formation as well as cell migration. On the other hand, TNFα increased the mature marker MAP2 along with the induction of neurite elongation, thus suggesting the ability of the cytokine to exert pro-differentiating effects on a more mature component of the cell culture able to explicate pro-survival mechanisms. Overall, our results may support the view that in the brain TNFα may exert both neurodegenerative and neuroprotective roles depending on other contributing factors, which mainly include the type of recruited receptors. Indeed, most of the negative proinflammatory functions are mediated by TNFR1, which is almost ubiquitously expressed by brain cells and it is mainly associated with neurodegeneration, while TNFR2, the highly regulated isoform primarily expressed at low levels by both immune cells and neurons [57], has been implicated in protective responses to promote neuronal survival. A recent study conducted in a mouse model of neurodegeneration demonstrated that co-administrating a TNFR1-antagonist with a TNFR2-agonist into the magnocellular nucleus basalis protected cholinergic neurons and their cortical projections against inflammatory insults, thus recovering memory impairing [58]. Here, we show that hfNBMs express both types of TNFα receptors and, accordingly to the literature, *TNFR1* was the most abundant, while *TNFR2* was expressed at very low levels. Interestingly, we found an increase of one order of magnitude of *TNFR2* mRNA expression in hfNBMs following TNFα stimulation, an effect that may suggest the induction of protective mechanisms. Although further studies are needed to fully elucidate the TNFR2-mediated neuroprotective pathways in hfNBMs, they could be associated with the pro-differentiating effects of TNFα that we observed in terms of increased MAP2 expression and neuritogenesis, most likely involving the mature component of the primary cell culture. 

Another important finding of this study was the identification of possible epigenetic mechanisms underlying TNFα action in hfNBMs, suggesting that TNFα may affect hfNBM maturation and phenotype by altering the DNA methylation status. In particular, we demonstrated that TNFα significantly increased the expression of DNMT1, the enzyme responsible for the maintenance of DNA methylation status. Our finding is consistent with previous works in other cell types that indicates an increase of this enzyme along with a hypermethylation of target promoters after TNFα stimulation [30,33,59,60]. Despite the well-known action of DNMT1 in the maintenance of DNA methylation status, recent studies also highlighted the role of overexpressed DNMT1 in de novo methylation [61,62]. To better investigate the occurrence of TNFα-mediated epigenetic mechanisms, here we analyzed the genome-wide DNA methylation pattern in hfNBMs, both at basal condition and after TNFα treatment. The global CpG methylation profile of untreated hfNBMs revealed high levels of methylation (average of 82.5%) comparable with previous studies on fetal brain cells [24,63,64] and no gross differences were observed with TNFα-treated samples. Although high methylation in non-CpG sites has been identified in adult neurons and embryonic stem cells [24,65], very low levels are detected in the fetal brain [66] and our results were consistent with these observations. The analysis of methylation levels was focused on the comparison of different genomic regions (CpG island, shore, promoter and gene body), mainly containing DNA methylation sites important for regulating gene transcription, in order to obtain a more sensitive detection of small differences, as expected during inflammatory stimulation [67]. Under the most stringent conditions (more than 20% significant differences in methylation levels between control and TNFα stimulated cells), we identified that 25 and 46 genomic elements with a significant methylation difference at the 24- and 48-h time points, respectively. A tendency to hyper-methylation was observed in TNFα DMRs at the 48-h time point, whereas at 24 h an equal distribution of hyper-methylated and hypo-methylated significant DMRs was observed. This preferential hyper-methylation at specific genomic elements detected only with the longer exposure to TNFα may be related to the marked increase of DNMT1. Indeed, the overexpression of *DNMT1* has been associated with selective promoter hyper-methylation and mRNA downregulation, as demonstrated in schizophrenic patients [68], as well as in the context of cancer biology, a pathological condition highly linked to inflammation [69]. The hypothesis that TNFα may alter the development of hfNBMs was confirmed by a functional enrichment analysis. The GO analysis of DMR-associated genes at 24 h of TNFα stimulation indicated an overrepresentation in developmental processes, regulation of transcription and different signaling pathways, including BMP signaling, which is important for neuronal development. Instead, at 48 h of inflammation most DMRs tended to be associated with genes strictly linked to nervous system development and regulation of transcription, suggesting the potential role of inflammation in interfering with NBM neuron development. Moreover, quantitative gene expression analysis confirmed a significant decrease in mRNA levels for *CHRDL1* and *MEST* genes, which showed a significantly hyper-methylated CpG island upon TNFα stimulus compared to untreated cells. Interestingly, both genes are implicated in neuronal development and migration. *MEST* is an imprinted gene encoding an α/β hydrolase which is expressed in both mesodermal derivatives and developing brain [70,71]. Although the role of this enzyme in the CNS is not fully understood, it functions as a modulator of the WNT/β-catenin pathway [72], an essential signal during brain development and in neurological diseases [73]. Moreover, recent work indicates an essential role of *MEST* for the development and maintenance of different neuronal subsets in the CNS [74,75]. Interestingly, the hyper-methylation of the *MEST* CpG island, as we reported after 48 h of TNFα stimulation, could regulate the expression of miR-335, a microRNA involved in fetal development and spatial memory as well as synaptic plasticity [76,77]. Similarly, *CHRDL1* is generally transcribed in migratory human neural progenitors and it is expressed in neurogenic regions [78]. Interestingly, Gaughwin and colleagues indicated that a reduction of *CHRDL1* transcript, due to epigenetic mechanisms, led to an increase of BMP-induced neurite outgrowth [79], and this finding correlates well with our data on a TNFα-induced decrease of CHRDL1 transcript and the increase of neurite outgrowth in hfNBMs. Overall, our findings strongly suggest an involvement of neuroinflammation in modulating hfNBMs developmental processes by epigenetic mechanisms.

## 4. Conclusions

In conclusion, in this work, we demonstrated that TNFα-driven inflammation affects the neuronal plasticity, maturation, and function of hfNBMs and suggest that changes in the DNA methylation status may be a relevant mechanism of action underlying TNF-α effects in human brain. In addition, our results strongly support a dual role of TNFα on NBM cholinergic neurons since it may exert a negative influence on neurogenesis despite a positive effect on differentiation, through mechanisms that remain to be elucidated and likely depending on the type of recruited receptor. It will be interesting to clarify whether manipulating TNFα signaling pathways dependent on its two distinct receptors could ameliorate the deficits of cholinergic neurons, neural circuits and behavioral functions. This could provide a potential therapeutic approach against neurodegenerative disorders.

## 5. Materials and Methods 

### 5.1. hfNBM Cell Culture

All the experiments were performed using hfNBM primary cell cultures previously obtained and characterized as already described [35] and used for experiments until passage 26. Briefly, brain biopsies from human fetuses (two 12-week old female fetuses) were obtained from voluntary abortions after the approval and sign of the informed consent document by the patient (ID: 20028/13 and 20029/13, released on 09/20/2013) according to the ethical guidelines of the Italian National Institute of Health as previously reported [80]. The NBM area was dissected under a stereomicroscope and digested with 1 mg/mL collagenase type IV (Worthington biochemical corp., Lakewood, NJ, USA; #4188). The cell suspension was mechanically dispersed by pipetting in Coon’s modified Ham’s F12 medium (Euroclone, Milan, Italy; #F6636) supplemented with 10% FBS (Hyclone, Logan, UT, USA; #SH30071.02). The cell suspension obtained was cultured in dishes at 37° C in 5 % CO_2_ atmosphere and routinely checked for mycoplasma contamination by PCR assay (mycoplasma plus™ PCR primer set, Agilent technologies, Santa Clara, CA, USA; #302008). The NBM cholinergic identity of hfNBMs was demonstrated by an extensive phenotypic and functional characterization previously described [35]. In brief, we demonstrated that hfNBMs express the major molecular markers of the cholinergic system, including proteins essential for Ach synthesis, transport, and hydrolysis (ChAT, VAchT, and AchE, respectively). Moreover, hfNBMs release Ach in the culture medium and express calbindin 1 (CALB1), along with both types of NGF receptors, TrkA and p75 NTR [35]. Indeed, the co-expression of CALB1 with both NGF receptors is consistent with the pattern described for cholinergic neurons in the human brain designed as “Ch4 neurons” by Mesulam and co-workers [81], who demonstrated that these neurons correspond to the NBM. Given the fetal origin, these cells also expressed immature markers, such as nestin, β-tubulin ΙΙΙ, and GFAP, and retained this phenotype for several passages in culture. Cells stored in liquid nitrogen and thawed after 1–12 months showed the same cholinergic phenotype as before storing. Cells were treated with TNFα 10 ng/mL for 3, 24, or 48 h to induce inflammation. NGF stimulation (100 ng/mL) were performed for 24 h in the presence or absence of TNFα (10 ng/mL). All experiments were performed in serum-free Coon’s modified Ham’s F12 medium (Euroclone) and cells were pre-serum-starved for 8 h, overnight or for 24 h. The use of human fetal biopsies for research purposes was approved by the National Ethics Committee and the local Ethics Committee for investigation in Humans of the University of Florence (Protocol Number: 678304, released on 02/01/2002).

### 5.2. Drugs and Antibodies

The recombinant human cytokine TNFα was purchased from Gibco (Gaithersburg, MD, USA; #PHC3016) and reconstituted in sterile distilled water following the manufacturers’ instructions. Human recombinant β-NGF was purchased from PeproTech Inc. (Rocky Hill, NJ, USA; #450-01) and reconstituted in culture medium. Drugs were stored at −20 °C as 10^2^ to 10^4^ times more concentrated stock solutions and dissolved at time of use in the medium culture to the final concentration. 

Primary antibodies for immunofluorescence, Western blot, and flow cytometry analysis included rabbit anti-ChAT polyclonal antibody (pAb; #AB143), rabbit anti-MAP2 pAb (#AB5622) from Merk Millipore (Temecula, CA, USA), rabbit anti-VAchT pAb (#SAB4200559), mouse anti-acetylated α-tubulin monoclonal antibody (mAb) from Sigma-Aldrich Corp. (St. Louis, MO, USA), mouse anti-α-tubulin mAb (#sc-8035), mouse anti-NF-κB p65 mAb (#sc-8008), rabbit anti-TrkA pAb (H-190; #sc-14024), mouse anti-DNMT1 mAb (H-12; #sc-271729), mouse anti-β-actin mAb (C-4; #sc-47778) from Santa Cruz Biotechnology (Santa Cruz, CA, USA) and mouse anti-p75 NTR mAb ACP-conjugated from Miltenyi Biotec (Bisley, Germany; ME20.4-1H4; #130-091-884). Alexa Fluor goat 488- or 568-conjugated secondary IgG antibodies (Molecular Probes, Eugene, OR, USA; #A11029 and #A11011, respectively) were used as appropriate for immunofluorescence and flow cytometry, whereas peroxidase-conjugated secondary IgG anti-mouse or anti-rabbit antibodies (Santa Cruz Biotechnology; #sc-2005 and #sc-2004, respectively) were used for Western blot analysis. ProLong Gold antifade reagent with DAPI (Invitrogen Corp., Carlsband, CA, USA; #p36941) was used to counterstain nuclei.

### 5.3. Immunofluorescence

Immunofluorescence analysis was performed as already published [82]. Briefly, hfNBMs were attached on sterile 20 × 20 mm coverslip, fixed with 2% paraformaldehyde in phosphate buffered saline (PBS; Sigma-Aldrich Corp.; #158127) for 10 min, permeabilized with 0.1% Triton X-100 (Sigma-Aldrich Corp.; #T8787) in PBS for 10 min and incubated with 1% bovine serum albumin (BSA; Sigma-Aldrich Corp.; #A2153) for 30 min at room temperature. Immunofluorescence staining was carried out using the following primary antibodies: anti-ChAT (1:200), anti-VAchT (1:1000), anti-acetylated α-tubulin (1:500), anti-α tubulin (1:2000) and anti-NF-κB p65 (1:100). Cells were next incubated with Alexa Fluor 488- or 568- conjugated secondary antibodies (1:200), as appropriate. Nuclei were counterstained using ProLong Gold antifade reagent with DAPI. Negative controls were obtained avoiding primary antibodies incubation. Neuritogenesis and primary cilium analysis were performed by counting stained cells with neurites longer than four times the cell body and the number of cells with a primary cilium, respectively. The neurite and cilia lengths were calculated using the ImageJ plugin NeuronJ (ImageJ, National Institute of Health, Rockville Pike, MD, USA, http://imagej.nih.gov/ij, 1.47t). All the measures were performed in blind by counting the stained cells in ten fields per slide of three different experiments. The evaluation of cilium length was performed in at least 15 cells for condition. Slides were imaged using the confocal microscopy Leica TCS SP5 (Leica Microsystems, Mannheim, Germany) or Nikon Microphot-FXA microscope (Nikon, Tokyo, Japan).

### 5.4. MTT Assay

Cell viability were evaluated by MTT assay (Sigma-Aldrich Corp.) as previously reported [36]. Briefly, hfNBMs were seeded at 8 × 10^3^ cells/well in 96 multi-well plates in culture medium (Coon’s modified ham’s F12 medium with 10% FBS). After 24 h, hfNBMs were serum-starved for 8 h and then stimulated with TNFα (10 ng/mL) for additional 24 h. Next, the medium was replaced and 10 μL of MTT solution was added for 3 h at 37 °C. The optical density was measured by a Multiscan FC spectrophotometer (Thermo Fisher Scientific, Waltham, MA, USA) with filter at 450 nm. Cell viability was reported as a percentage of untreated cells, taken as 100% (mean ± SEM). Each experimental point was in quadruplicate and three different experiments were performed to obtain statistical significance.

### 5.5. RNA Extraction and Quantitative RT-PCR Analysis

Total RNA extraction from 2 × 10^5^ hfNBMs was carried out using the “RNeasy Micro kit” (Qiagen, Hilden, Germany; #74004) according to the manufacturers’ instructions. cDNA synthesis was performed by the iScript^TM^ cDNA Synthesis Kit from Bio-Rad Laboratories (Hercules, CA, USA; #1708891). Quantitative real time RT-PCR (qRT-PCR) was performed for some genes according to the fluorescent TaqMan method, as already reported [83]. Probes and primers specific for target genes were predeveloped assays (Life Technologies, Carlsbad, CA, USA; Supplementary Table S1). Genes were analyzed by qRT-PCR using SsoAdvancedTM Universal SYBR® Supermix and a CFX96 Two-Color Real-Time PCR Detection System (Bio-Rad Laboratories; #1725271) with the following thermal cycler conditions: 40 cycles at 95 °C for 15 secs and 60 °C for 1 min. Specific primer sequences for qRT-PCR were custom made using sequences available at NCBI GenBank (http://www.ncbi.nlm.nih.gov; accessed on 02/06/2018) or Ensembl Genome (http://www.ensembl.org; accessed on 02/06/2018) and are reported in Supplementary Table S2. *18S* ribosomal RNA subunit was chosen as housekeeping gene and its expression was quantified with a predeveloped assay (Life Technologies; Hs99999901_s1) and used during the analysis for relative quantification of the target genes. Data analysis was carried out using the comparative threshold cycle (Ct) with 2^−ΔΔCt^ method as already reported [84]. 

### 5.6. Western Blot Analysis

The protein extracts were obtained in RIPA lysis and extraction buffer (Thermo Fisher Scientific; #89900) supplemented with protease inhibitors cocktails (1:100; Sigma-Aldrich Corp.; #P8340) following the manufacturers’ instruction and quantified using Coomassie protein assay kit (Bio-Rad Laboratories; #500-0006). Aliquots containing 20 µg of protein extract were subjected to immunoblotting as previously described [82]. Briefly, proteins were loaded on SDS-PAGE, next transferred on polyvinylidene difluoride membranes (GE Healthcare, Little Chalfont, UK; #10600023) and blocked in 3% BSA in PBS. Specific antibodies for protein detection were used according to the manufacturer’s suggested dilution range as follows: anti-MAP2 (1:500) and anti-TrkA (1:1000), anti-DNMT1 (1:500), and anti-β-actin (1:10000). Peroxidase-conjugated secondary IgG anti-mouse or anti-rabbit antibodies at 1:5000 dilution in PBS were used, as appropriate. The proteins were revealed by the enhanced chemiluminescence system LiteAblot extend (Euroclone; #EMP013001). Image acquisition was performed with Quantity One software on a ChemiDoc XRS instrument (Bio-Rad Laboratories) or with Amersham Hyperfilm (GE Healtcare, Milan, Italy) and densitometric analysis was performed by open source Java-based ImageJ analysis software (https://imagej.net/). β-actin protein was used for protein normalization. Three different sets of experiments were performed to obtain statistical significance.

### 5.7. Flow Cytometry

hfNBMs were analyzed by flow cytometry as already described [41]. Briefly, 2 × 10^5^ hfNBMs were resuspended in PBS supplemented with 1% FBS and, after fixation with paraformaldehyde 2% in PBS, incubated with anti-TrkA (1:100) or anti-p75 NTR ACP-conjugated (1:20) primary antibodies. Alexa Fluor 488 goat anti-mouse IgG (1:200) was used as secondary antibody for anti-TrkA. Negative controls were obtained avoiding primary antibodies incubation, while autofluorescence was evaluated in cells without antibodies. Stained cells were analyzed on a FACSCanto II instrument (BD Pharmingen, San Diego, CA, USA). Data were analyzed using BD FACSDiva Software (BD) and FlowJo v10 (Tree Star, Inc., Ashland, OR, USA).

### 5.8. DNA Extraction and Reduced Representation Bisulfite Sequencing (RRBS) Library Preparation

The whole genome methylation analysis was performed by reduced representation bisulfite sequencing (RRBS) technique [85,86]. Genomic DNA was extracted from 5 × 10^5^ cells from biological triplicates of TNFα treated and untreated hfNBMs for each experimental time points (24 and 48 h) by QIAamp® Blood Mini Kit (Qiagen; #51104) following the manufacturer’s instructions. Extracted DNA was processed in order to perform RRBS analysis. Genomic DNA was digested with MspI restriction enzyme (Fermentas, Vilinius, Lithuania; #ER0541), followed by end-repair and T-tailing using Klenow Fragment 3’→5’ exo- (New England Biolabs, Beverly, MA, USA; #M0212). Unique molecular identifier (UMIs) method was performed to ligate cytosine methylated TruSeq adapters (Illumina Inc, San Diego, CA, USA; #ME100-0010). Ligated DNA was next purified by AMPure XP magnetic beads (Beckman Coulter Genomics, Chaska, MN, USA; #A63882) and size selected. Bisulfite conversion, desulphonation and purification were performed using EZ DNA methylation direct kit (Zymo research, Irvine, CA, USA; #D5020) as per the manufacturer’s protocol. Each amplified DNA library was purified by AMPure magnetic XP beads (Beckman Coulter Genomics) and run with high sensitivity DNA chip on a 2100 Bioanalyser (Agilent Technologies, Santa Clara, CA, USA) to assess quality and fragment sizes of RRBS libraries. Quantitation was performed by KAPA library quantification kit (Hofmann-La Roche, Basel, Switzerland; #KK4824) and all RRBS libraries were sequenced by single-end (100 bp) sequencing in one lane of the Illumina HiSeq2000 platform, as per the manufacturer’s recommendations. 

### 5.9. DNA Methylation Data Processing 

Raw sequencing data (Fastq files) were processed for quality by Fast QC program version 0.11.5 (https://www.bioinformatics.babraham.ac.uk/projects/fastqc/; Babraham Bioinformatics, Cambridge, UK). The raw sequenced reads were initially processed to remove the first 13 bp containing the UMI sequencing tags and next trimmed for adapters and poor-quality bases with the wrapper script Trim Galore! version 0.4.4 (https://www.bioinformatics.babraham.ac.uk/projects/trim_galore/; Babraham Bioinformatics). Bismark version 0.18.2 [87] was used to align trimmed reads to the human genome (GRCh38 build) using --directional option with default parameters in single-end mode and UmiBam (https://github.com/FelixKrueger/Umi-Grinder/blob/master/UmiBam; v0.0.1, Felix Krueger, Babraham Bioinformatics) was used to remove duplicated reds. The UMI deduplicated reads were processed with Bismark Methylation Extractor (Babraham Bioinformatics) using the default parameters to generate reports with content-specific methylation information. CpG methylation calls were analyzed and visualized by SeqMonk Mapped Sequence Data Analyser (https://www.bioinformatics.babraham.ac.uk/projects/seqmonk/; v1.38.2, Babraham Bioinformatics) with R software implementation (https://cran.r-project.org/bin/windows/base/; v3.5.1, The R Foundation for Statistical Computing, Institute for Statistics and Mathematics, Wien, Austria). Three replicates for each experimental condition were analyzed at two different time points (24 and 48 h) independently. Triplicates were used as opposed to pool data to improve the power rather than the coverage of the analysis [88]. Whole genome methylation analysis was performed at different genomic features as follows: CpG islands (list of CpG islands from the SeqMonk feature annotation table; *n* = 22,564), shores (regions 2kb upstream and 2kb downstream CpG islands; *n* = 45,128), promoters (regions 2kb upstream Transcription Start Site of the genes from the SeqMonk feature annotation table; *n* = 153,967) and gene bodies (list of gene bodies from the SeqMonk feature annotation table; *n* = 60,099). The SeqMonk tables refers to Ensembl annotation. Only genomic features elements with ≥5 CpGs with read count ≥3 in all the replicates were considered for downstream analysis. Methylation level (i.e., percentage of methylation or beta value) was obtained by averaging the methylation level of CpG sites mapping to each element using SeqMonk. When the methylation level was compared, DMRs within the first 30 percentile of normalized read count (i.e., by total read count) were filtered out to obtain a reasonable CpGs measure in all the replicates. Statistically significant differences were computed by the logistic regression method [89] and p-values were corrected for multiple testing using Benjamini–Hochberg correction (FDR) with ratios recalculated from normalized quantitation. Significant DMRs were identified those showing ≥10% or ≥20% difference in methylation level and FDR <0.05. Gene annotation of significant DMRs were performed using the “closest to gene” utility of SeqMonk (using annotation distance cut-off of 2kb). Functional analysis of significant DMRs was performed by DAVID Bioinformatics Resources version 6.8 (https://david.ncifcrf.gov; Frederick National Laboratory for Cancer Research, Frederick, MD, USA)

### 5.10. Methylation-Sensitive Restriction Analysis

RRBS results on methylation level for *CHRDL1* promoter gene were validated by methylation-sensitive restriction qPCR analysis using the EpiTect Methyl II PCR assay (Qiagen; #335002) for human *CHRDL1* CpG Island (115193-GRch37, UCSC genome), corresponding to the region of interest. Serum-starved hfNBMs (6 × 10^5^) were treated with TNFα for 48 h and DNA was extracted by QIAamp® Blood Mini Kit (Qiagen) following the manufacturer’s instructions. The DNA extracts from control (untreated cells) and TNFα treated groups were next digested and prepared for the methylation analysis according to the manufacturer’s instructions. The residual DNA generated by each individual enzyme reaction was quantified by real time qPCR using a Rotor-Gene 6000 (Qiagen) with primers flanking the restriction sites of *CHRDL1* CpG island of interest. qPCR protocol was performed following the standard amplification conditions reported on product instructions using RT qPCR SYBR Green MasterMix (Qiagen; #330500). In order to verify the cutting enzyme efficiency, we used SEC and DEC assays (Qiagen; #EPHS115450-1A and #EPHS115451-1A, respectively). The values processed were reliable only if SEC and DEC passed the quality control. Differences in DNA methylation level were analyzed using the supplied EpiTect Methyl II PCR Microsoft Excel template. *CHRDL1* methylation analysis was performed in duplicates on three independent experiments. 

### 5.11. Migration Assay

Cell migration was assessed using a 48-well chemotaxis chamber (Neuro Probe, Cabin John, MD, USA) with polycarbonate membranes (pore size 8 µm; Neuro Probe; #PFB8) coated with collagen type I (20 µg/mL, Sigma-Aldrich Corp.; #CC050). Briefly, the lower wells of the chamber were filled with serum-free or 10% FBS culture medium to evaluate basal or induced hfNBM migration, respectively. TNFα pre-treated (10 ng/mL for 48 h) or untreated hfNBMs (4 × 10^4^ cells) were seeded in serum starved condition into the upper well and incubated for 6 h at 37 °C in 5% CO_2_ atmosphere. After the incubation period, the migrated cells in the lower side of the membrane were fixed with absolute methanol for 15 min, washed with PBS, and stained for 30 min with 10% Giemsa solution (BioOptica, Milan, Italy; #05-12005E) in PBS. No migrated cells, in the upper part of the membrane, were removed and the filter was mounted on a glass slide for visualization. The number of migrated hfNBMs was counted in blind under an optical microscope (Zeiss Axioskop 20; Carl Zeiss S.p.A., Milan, Italy) in 10 fields for each well. Each experimental point was replicated at least six times in three independent experiments.

### 5.12. Statistical Analysis

Data were expressed as mean ± standard error of mean (SEM). Student’s unpaired *t*-tests or one-way ANOVA followed by Tukey’s post-hoc analyses for multiple comparison were performed, as appropriate, in order to determine statistical significance which was defined as *p* < 0.05. Data were analyzed using the Statistical Package for the Social Sciences (SPSS v.25.0; SPSS Inc., Chicago, IL, USA; https://www.ibm.com/support/pages/downloading-ibm-spss-statistics-25). 

Whole genome methylation analysis were performed and visualized by SeqMonk Mapped Sequence Data Analyser v1.38.2 (https://www.bioinformatics.babraham.ac.uk/projects/seqmonk/; Babraham Bioinformatics) and R software (https://cran.r-project.org/bin/windows/base/; The R Foundation for Statistical Computing). The logistic regression method [89] was used in order to determine the statistical significance and p-values were corrected for multiple testing using Benjamini–Hochberg correction (FDR < 0.05) with ratios recalculated from normalized quantitation.

## Figures and Tables

**Table 1:** List of genes proximal to significant DMRs with a minimum difference of 20%.

						*% of Methylation*		
	Chr	Associated Gene	ID	Genomic Element	FDR	CTL	TNFα	TNFα Methylation Level vs CTL
24-hour time point	6	*PLAGL1*	ENSG00000118495	CpG island	5.41 × 10^−41^	60.49	91.14	**hypermethylated**
				Promoter	2.06 × 10^−15^	60.74	91.11	**hypermethylated**
	20	*HSPA12B*	ENSG00000132622	CpG island	2.57 × 10^−19^	11.11	31.43	**hypermethylated**
	17	*KSR1*	ENSG00000141068	Promoter	5.15 × 10^−4^	25	53.89	**hypermethylated**
	X	*LAMP2*	ENSG00000005893	CpG island	1.95 × 10^−6^	48.74	69.95	**hypermethylated**
				Gene body	1.98 × 10^−6^	48.74	69.95	**hypermethylated**
	X	*NR0B1*	ENSG00000169297	CpG island	4.13 × 10^−17^	81.35	57.14	**hypomethylated**
				Promoter	5.56 × 10^−17^	81.35	57.14	**hypomethylated**
				Gene body	2.00 × 10^−19^	81.35	57.14	**hypomethylated**
	1	*MEGF6*	ENSG00000162591	Shore	1.10 × 10^−5^	86.67	63.33	**hypomethylated**
	4	*SOWAHB*	ENSG00000186212	Shore	0.00123	65.7	43.33	**hypomethylated**
				Gene body	1.98 × 10^−6^	65.7	43.33	**hypomethylated**
	16	*MSLN*	ENSG00000102854	Shore	1.58 × 10^−7^	77.55	55.61	**hypomethylated**
	8	*PVT1*	ENSG00000249859	Promoter	5.15 × 10^−4^	50	26	**hypomethylated**
48-hour time point	7	*MEST*	ENSG00000106484	CpG island	8.53 × 10^−18^	62.42	82.53	**hypermethylated**
				Promoter	7.28 × 10^−7^	62.42	82.53	**hypermethylated**
	14	*GSC*	ENSG00000133937	CpG island	5.35 × 10^−9^	7.78	32.59	**hypermethylated**
				Shore	1.96 × 10^−9^	7.78	32.59	**hypermethylated**
				Promoter	2.16 × 10^−8^	7.78	32.59	**hypermethylated**
	X	*CHRDL1*	ENSG00000101938	CpG island	8.00 × 10^−5^	35	60.42	**hypermethylated**
				Promoter	5.11 × 10^−5^	35	60.42	**hypermethylated**
	X	*DOCK11*	ENSG00000147251	CpG island	1.25 × 10^−11^	48.61	80.95	**hypermethylated**
	X	*ARMCX2*	ENSG00000184867	Promoter	7.46 × 10^−5^	31.27	52.26	**hypermethylated**
	22	*RPL3*	ENSG00000100316	Promoter	0.00384	46.67	67.78	**hypermethylated**
	1	*TRIM62*	ENSG00000116525	Gene body	3.85 × 10^−8^	39.17	63.89	**hypermethylated**
	8	*XKR6*	ENSG00000171044	Gene body	0.00243	40.71	65.56	**hypermethylated**
	X	*ARMCX2*	ENSG00000184867	Promoter	5.11 × 10^−5^	31.27	52.26	**hypermethylated**
	X	*DCAF12L2*	ENSG00000198354	Promoter	1.18 × 10^−35^	47.37	87.68	**hypermethylated**
	20	*MYBL2*	ENSG00000101057	Shore	5.09 × 10^−4^	71.43	46.22	**hypomethylated**
				Promoter	5.09 × 10^−4^	71.43	46.22	**hypomethylated**
	16	*PRDM7*	ENSG00000126856	Promoter	7.98 × 10^−6^	65.07	43.6	**hypomethylated**
	21	*TFF2*	ENSG00000160181	Promoter	2.06 × 10^−4^	71.78	50.21	**hypomethylated**
	22	*HDAC10*	ENSG00000100429	Promoter	1.24 × 10^−4^	65.33	44.76	**hypomethylated**
	X	*WAS*	ENSG00000015285	Promoter	1.85 × 10^−6^	63.22	41.92	**hypomethylated**
	14	*TMEM179*	ENSG00000258986	Gene body	2.08 × 10^−5^	70.64	49.31	**hypomethylated**

TNFα methylation level vs. CTL is shown in red (hypermethylated) or green (hypomethylated).

## Supplementary Materials

Supplementary materials can be found at https://www.mdpi.com/1422-0067/21/17/6128/s1.

## Abbreviations

hfNBMs	human fetal nucleus basalis of Meynert cells
TNFα	tumor necrosis factor α
CNS	central nervous system
ChAT	choline acetyltransferase
VAchT	vesicular acetylcholine transporter
COX2	cyclooxygenase 2
GFAP	glial fibrillary acidic protein
AD	Alzheimer’s disease
PD	Parkinson’s disease
BFCNs	basal forebrain cholinergic neurons
NBM	nucleus basalis of Meynert
NGF	nerve growth factor
IFT88	intraflagellar Transport 88
TrkA	tropomyosin receptor kinase A
p75 NTR	neurotrophin Receptor p75
CALB1	calbindin 1
qRT-PCR	quantitative real time RT-PCR
PBS	phosphate buffered saline
FBS	fetal bovine serum
RRBS	reduced representation bisulfite sequencing
UMIs	unique molecular identifiers
DMRs	differentially methylated regions
GO	Gene Ontology
